# Remazolam on postoperative cognitive dysfunction: A perspective

**DOI:** 10.1097/MD.0000000000041995

**Published:** 2025-05-02

**Authors:** Liang Zhang, Qing-Hua Wang, Yi Qiu, Yu-Mei Ding, Xiao-Dong Wang, Zhi-Feng Zhang, Yi-Fan Zhao, Liang-Liang He

**Affiliations:** a Department of Anaesthesiology, The Second Affiliated Hospital of Inner Mongolia Medical University, Hohhot, China; b Department of Anaesthesiology, People’s Hospital of Ordos Dongsheng District, Ordos, China; c Department of Joint Surgery, The Second Affiliated Hospital of Inner Mongolia Medical University, Hohhot, China; d School of Public Health, Inner Mongolia Medical University, Hohhot, China; e Department of Pain Management, Xuanwu Hospital, Capital Medical University, Beijing, China.

**Keywords:** association, pathogenesis, pharmacological action, postoperative cognitive dysfunction, remazolam

## Abstract

This review thoroughly examines the complex interplay between remazolam administration and postoperative cognitive dysfunction (POCD), offering a detailed analysis of POCD’s pathogenesis and the pharmacological mechanisms underpinning remazolam’s effects. It critically synthesizes existing research findings, highlighting the nuanced nature of the association between remazolam use and POCD. While recognizing the widespread use of remazolam as a sedative, this review identifies its potential to increase susceptibility to POCD, particularly in vulnerable groups such as elderly patients or those exposed to prolonged or high-dose administration. However, it underscores the variability in research outcomes, necessitating a deeper exploration. The review underscores the necessity for tailored guidelines on remazolam administration and advocates for holistic POCD prevention strategies, integrating pharmacological measures with complementary non-pharmacological approaches to enhance patient safety. The review emphasizes the need for rigorous future research, including randomized controlled trials and longitudinal studies, to unravel the intricate dynamics between remazolam use and POCD, providing clinicians with robust evidence to guide optimal patient care.

## 1. Introduction

Postoperative cognitive dysfunction (POCD) is a complex condition characterized by impairments in cognitive domains such as memory, attention, information processing speed, and executive functions, observed both in the immediate and long-term periods following surgical and anesthetic procedures.^[1]^ POCD significantly detracts from patients’ quality of life, prolongs recovery periods, and is potentially linked to enduring neurocognitive deficits, predominantly affecting the elderly population.^[2]^ The etiology of POCD remains multifaceted and not entirely elucidated, but prevailing evidence points to factors such as systemic inflammatory responses triggered by surgery, the pharmacological effects of anesthetic agents, patient-specific variables including age and preexisting health conditions, and intraoperative hemodynamic fluctuations.^[3,4]^

Remazolam, a benzodiazepine sedative favored for its rapid onset and brief duration of effect, alongside a notable safety profile, is extensively employed in the facilitation of surgical anesthesia and sedation. Its mechanism involves the potentiation of γ-aminobutyric acid (GABA) receptor activities in the central nervous system, culminating in sedative and hypnotic outcomes.^[5]^ Recent discourse on POCD has increasingly scrutinized the implications of remazolam, especially given observations that its administration may correlate with cognitive declines postsurgery, especially among the elderly and those receiving it in high doses or over prolonged periods. Nevertheless, the literature presents a mixed picture due to variability in research design, sample sizes, and cognitive assessment methodologies, which together challenge the consistency and veracity of findings.^[6]^

The investigation into the linkage between remazolam administration and POCD is deemed critically important for clinical practice.^[7]^ It not only promises to shed light on the underlying mechanisms predisposing to POCD, thereby laying the groundwork for preventive and therapeutic modalities but also informs the judicious selection and application of anesthetic agents to mitigate the risk of cognitive impairments postsurgery.^[8]^ This review is dedicated to a comprehensive review of existing studies on the association between remazolam use and POCD, with an emphasis on dissecting the current understanding, identifying potential relationships and influencing variables, and proposing directions for future research and clinical applications.

## 2. Search strategy and selection criteria

### 2.1. Search strategy

A comprehensive and systematic search was conducted to identify relevant literature exploring the effects of remazolam on POCD. The search strategy included a combination of key terms such as “remazolam,” “Byfavo,” “postoperative cognitive dysfunction,” “postoperative cognitive decline,” “postsurgical cognitive decline,” “cognitive dysfunction after surgery,” and “surgical cognitive impairment.” Searches were performed in the China National Knowledge Infrastructure and PubMed databases, encompassing studies published from their inception to March 2024. Both English and Chinese language publications were considered to maximize the inclusivity of the review.

Additionally, manual searches of reference lists from relevant studies were performed to ensure no pertinent studies were omitted.

### 2.2. Inclusion criteria

Studies were considered eligible for inclusion based on the following criteria: the primary focus was on the effects of remazolam on POCD, either in clinical or preclinical settings; studies presented measurable clinical outcomes, experimental evidence, or biomarker analyses directly evaluating the impact of remazolam on cognitive function following surgery; the studies were peer-reviewed and published in recognized journals in English or Chinese languages; and both randomized controlled trials and observational studies that provided robust data on remazolam’s efficacy or safety in the context of POCD were included.

### 2.3. Exclusion criteria

Studies meeting any of the following conditions were excluded: studies that did not directly address the role of remazolam in POCD or primarily focused on interventions unrelated to remazolam; articles that were duplicate reports or reviews without original data to avoid redundancy; studies lacking sufficient data or outcomes relevant to POCD; and gray literature, conference abstracts, or unpublished studies not subject to peer review to maintain academic rigor.

### 2.4. Study selection

The study selection process was conducted in 3 distinct phases to ensure methodological rigor and consistency. First, 2 independent authors screened the titles and abstracts of all retrieved studies against the predefined inclusion and exclusion criteria. Next, studies that passed the initial screening underwent a detailed full-text review to confirm eligibility. Finally, discrepancies between reviewers were resolved through discussion, and when consensus could not be reached, a third author was consulted to make the final determination. This process ensured objectivity and minimized potential bias. The inclusion of a dual-reviewer system and consultation with a third author.

**Figure 1.**
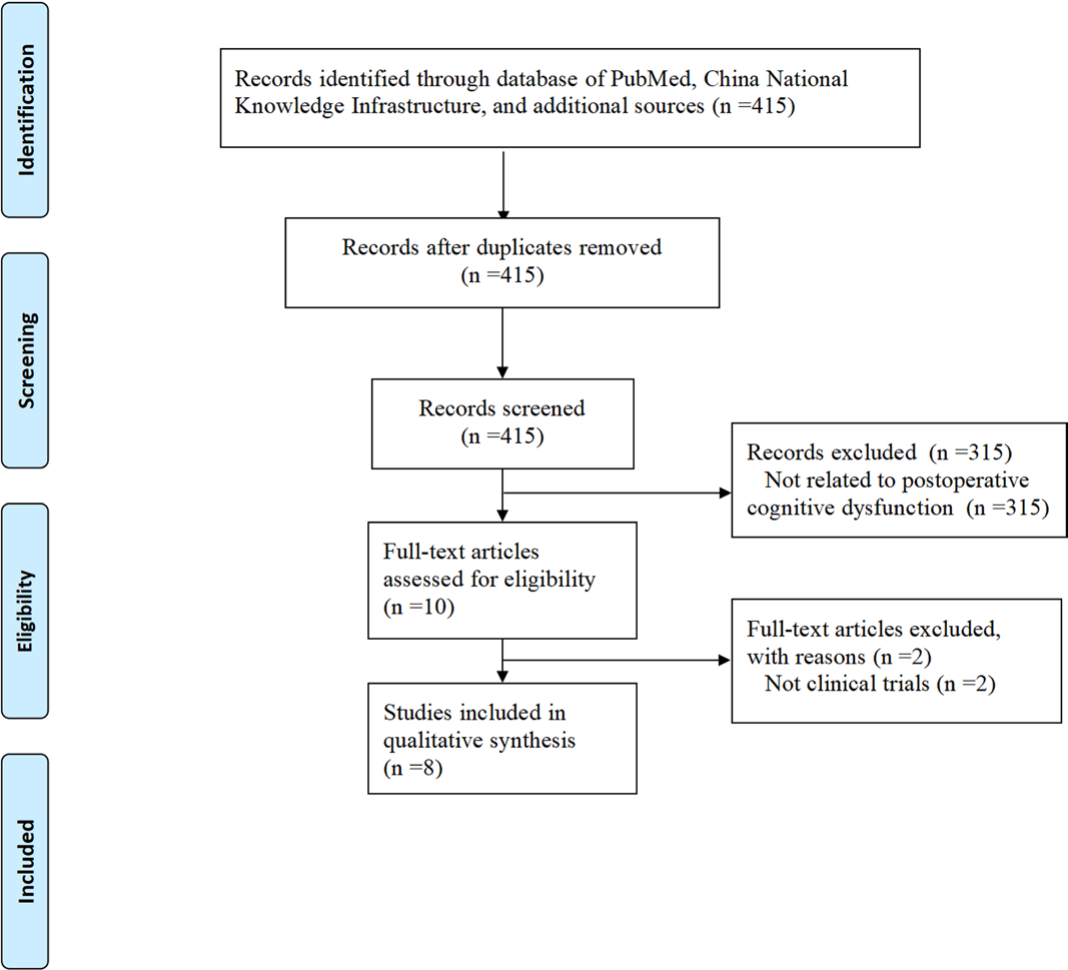
Flowchart of study selection.

A total of 415 records were initially identified through a comprehensive search. After a meticulous screening process, 315 studies unrelated to POCD were excluded (Fig. 1). Subsequently, the full texts of the remaining 10 studies were thoroughly assessed for eligibility based on predefined inclusion criteria. Of these, 8 clinical trials were ultimately included in the study, while 2 were excluded as they did not qualify as clinical trials (Fig. 1). This stepwise process ensures the selection of relevant and high-quality evidence for analysis.

## 3. Pharmacological insights into remazolam and POCD

Remazolam has gained recognition for its distinctive pharmacological features, including a rapid onset and short duration of action. These characteristics make it an attractive choice for surgical anesthesia and critical care, offering effective sedation with minimal residual effects. However, its mechanism of action, potentiating GABA receptor activity, has raised concerns about its potential role in POCD, particularly in vulnerable populations such as elderly patients or those subjected to prolonged or high-dose administration.^[5,9]^

Structurally, remazolam is designed to optimize efficacy and safety. Modifications to its molecular functional groups enhance its pharmacokinetics, enabling rapid absorption and a reduced half-life.^[9]^ These attributes facilitate precise control of sedation levels while minimizing residual drug accumulation, making remazolam particularly suitable for surgical procedures requiring short-acting anesthetics. The drug’s pharmacodynamic profile also supports its use in outpatient settings where rapid recovery is critical.

The primary mechanism of remazolam’s action involves the activation of GABA receptors, which are the predominant inhibitory neurotransmitters in the central nervous system. This activation promotes the opening of chloride ion channels, leading to neuronal membrane hyperpolarization and a reduction in excitatory neural activity.^[5]^ These effects not only provide pronounced sedative and hypnotic outcomes but also complement other anesthetic regimens, significantly reducing patient anxiety during preoperative preparation.

Clinically, remazolam’s rapid onset and short half-life offer notable benefits, allowing healthcare providers to tailor sedation levels to individual patient needs. Its predictable pharmacokinetics enhance safety and facilitate faster recovery, particularly in patients undergoing outpatient surgeries or minor procedures.^[10]^ These characteristics contribute to its increasing preference among anesthesiologists for diverse procedural contexts.

Despite its advantages, remazolam’s use is not without risks. Prolonged or excessive administration can lead to tolerance, dependence, and suppression of central nervous system activity.^[9]^ These risks are especially relevant in elderly patients, who are more susceptible to POCD due to age-related neurophysiological changes and increased sensitivity to sedatives.^[9]^ Evidence suggests that extended or high-dose exposure to remazolam may exacerbate neuronal suppression, heightening the risk of cognitive impairments in this population.^[5,9]^

The dual-edged nature of remazolam’s pharmacological effects underscores the need for careful clinical application. Individualized dosing strategies are essential to mitigate the risk of adverse outcomes. Healthcare providers should consider factors such as patient age, baseline cognitive status, and comorbidities when administering remazolam. Furthermore, future research is crucial to elucidate the precise relationship between remazolam and POCD, focusing on high-risk populations and varying dosage regimens to optimize clinical practice.

## 4. Pathogenesis of POCD

POCD is a multifactorial condition characterized by transient or prolonged impairments in cognitive function following surgical procedures. The underlying mechanisms of POCD involve a complex interplay between systemic inflammatory responses, neuroinflammation, oxidative stress, and disruption of the blood–brain barrier (BBB).^[11]^ These interconnected processes contribute to neuronal damage, synaptic dysfunction, and subsequent cognitive decline, particularly in vulnerable populations such as the elderly.

Surgical trauma triggers a systemic inflammatory response, marked by the release of pro-inflammatory cytokines, including interleukin-6 (IL-6) and tumor necrosis factor-alpha.^[4]^ These cytokines can cross the BBB, inducing neuroinflammation that impairs neuronal function and cognitive processes. Elevated levels of postoperative inflammatory markers have been closely associated with the incidence and severity of POCD, underscoring the critical role of systemic inflammation in its pathogenesis.^[4]^ Furthermore, the activation of microglia, the brain’s resident immune cells, plays a central role in neuroinflammation. Once activated, microglia release inflammatory mediators and reactive oxygen species, which exacerbate neuronal damage and disrupt synaptic transmission.^[12]^

Oxidative stress is another pivotal factor in POCD pathogenesis. Reactive oxygen species generated during surgical procedures and anesthetic administration target cellular components such as lipids, proteins, and DNA, resulting in oxidative damage and apoptosis.^[13]^ The brain’s high metabolic rate and lipid-rich environment make it particularly susceptible to oxidative damage, further contributing to cognitive deficits. Additionally, surgical stress and anesthetic agents can compromise BBB integrity, allowing the infiltration of inflammatory mediators and neurotoxins into the brain parenchyma.^[14]^ This breach not only amplifies neuroinflammatory responses but also disrupts homeostatic processes critical for cognitive function.

Anesthetic agents themselves may have direct effects on neuronal activity and neurotransmitter dynamics, further complicating POCD’s etiology. These agents can alter synaptic transmission and impair neuroplasticity, while their impact on cerebral perfusion and metabolism may exacerbate neuronal dysfunction.^[15]^ Combined with the systemic effects of surgery, these direct actions highlight the multifactorial nature of POCD.

The pathogenesis of POCD reflects a complex and dynamic interplay of systemic inflammation, oxidative stress, BBB disruption, and direct effects of anesthetic agents.^[2,4]^ These mechanisms vary between individuals and are influenced by factors such as age, preexisting cognitive status, and comorbidities. This heterogeneity underscores the necessity for tailored perioperative strategies to mitigate POCD risk and enhance patient outcomes. Addressing these interconnected pathways through targeted interventions holds promise for reducing the cognitive burden associated with surgical procedures.

## 5. Current evidence and clinical implications of remimazolam in POCD

Emerging evidence underscores the advantages of remimazolam as an anesthetic agent in minimizing POCD, particularly among elderly surgical patients. The following discussion synthesizes current evidence and clinical implications, presenting a refined analysis^[16–23]^ (Table 1).

**Table 1:** Summary of included studies.

Reference	Publication type	Patients	Intervention	Main findings
Kuang Q, et al (2023)^[16]^	Randomized Controlled Trial	Older patients undergoing pulmonary lobectomy	Propofol versus remimazolam	Remimazolam provided better hemodynamic stability and cognitive outcomes during one-lung ventilation compared to propofol.
Wu XQ (2024) ^[17]^	Randomized Controlled Trial	Elderly orthopedic patients	Propofol versus remimazolam	Remimazolam maintained more stable cerebral oxygen saturation and hemodynamics but showed no significant difference in POCD incidence compared to propofol.
Chen QQ, et al (2024)^[18]^	Randomized Controlled Trial	Elderly patients undergoing endoscopic sinus surgery	Propofol versus remimazolam	Remimazolam improved cerebral oxygen saturation and reduced inflammatory markers (serum S100β and IL-6) compared to Propofol, but did not significantly affect POCD in elderly sinus surgery patients.
Yang YP, et al (2023)^[19]^	Randomized Controlled Trial	Elderly patients undergoing painless gastrointestinal endoscopy	Propofol versus remimazolam	Remimazolam had fewer side effects, better hemodynamic stability, and no significant increase in POCD risk compared to propofol.
Liu BW (2023)^[20]^	Randomized Controlled Trial	Elderly patients undergoing total hip replacement	Remimazolam versus midazolam	Remimazolam reduced inflammatory factors (serum S100β, IL-6) and provided faster recovery with fewer side effects compared to midazolam.
Shi NH (2023)^[21]^	Randomized Controlled Trial	Elderly patients undergoing hip replacement surgery	Remimazolam versus midazolam	Remimazolam improved recovery quality and reduced POCD and agitation incidence compared to midazolam.
Zhu WX (2022)^[22]^	Randomized Controlled Trial	Elderly patients undergoing orthopedic surgery	Remimazolam versus propofol	Remimazolam resulted in better cognitive outcomes, faster recovery, and more stable hemodynamics compared to propofol.
Chen QQ (2022)^[23]^	Observational Study	Elderly patients undergoing endoscopic sinus surgery	Remimazolam versus propofol	Remimazolam reduced inflammatory markers and improved recovery quality with no significant difference in POCD compared to propofol.

POCD = postoperative cognitive dysfunction.

Hemodynamic stability: remimazolam demonstrates superior hemodynamic stability compared to conventional sedatives like propofol and midazolam. This stability is critical for elderly patients, who are often more susceptible to cardiovascular complications. By maintaining consistent blood pressure and heart rate, remimazolam reduces perioperative risks and helps preserve cerebral perfusion, which is essential for minimizing cognitive decline^[16,17]^ (Table 1). Such stability plays a pivotal role in reducing the risk of intraoperative and postoperative complications that could adversely affect cognitive function.

Cerebral oxygenation: one of the key benefits of remimazolam is its ability to maintain higher levels of intraoperative cerebral oxygen saturation compared to propofol. Enhanced intraoperative cerebral oxygen saturation indicates better oxygen delivery to the brain, which is crucial for neuroprotection during surgery. This effect reduces the incidence of cerebral desaturation events, a known risk factor for POCD, thereby promoting better postoperative cognitive outcomes^[18,23]^(Table 1).

Anti-inflammatory effects: remimazolam has been shown to significantly reduce levels of inflammatory markers such as S100β and IL-6, both of which are closely linked to neuroinflammation and the pathogenesis of POCD. By mitigating inflammatory responses during and after surgery, remimazolam offers a protective effect against neurocognitive decline, adding an important dimension to its clinical utility^[19,20]^ (Table 1).

Recovery and cognitive outcomes: studies consistently report that remimazolam facilitates faster recovery and improved early postoperative cognitive function compared to midazolam and propofol. Patients treated with remimazolam experience shorter recovery times, reduced agitation, and fewer incidences of early POCD. These benefits not only enhance patient outcomes but also reduce the burden on healthcare systems by expediting recovery and minimizing postoperative complications^[21,22]^ (Table 1).

Clinical implications: the evidence supports remimazolam as a preferred sedative for elderly patients, who are at heightened risk of POCD due to age-related vulnerabilities. Its applicability across various surgical settings, ranging from orthopedic procedures to gastrointestinal and pulmonary surgeries, further highlights its versatility. The combination of hemodynamic stability, improved cerebral oxygenation, reduced inflammation, and faster recovery positions remimazolam as a promising agent for perioperative neuroprotection. This potential warrants further research to investigate its long-term effects and underlying mechanisms.

In summary, remimazolam offers a multifaceted approach to reducing the risk of POCD and improving perioperative outcomes in elderly surgical patients. By addressing hemodynamic, neuroinflammatory, and recovery-related challenges, it presents a significant advancement in anesthetic practice. Future studies should focus on its long-term cognitive effects and explore its broader application in high-risk populations.

## 6. Discussion

This study highlights the significant advantages of remazolam over conventional sedatives in managing POCD. Key findings include its superior hemodynamic stability, reduced recovery time, fewer adverse events, and potential neuroprotective effects, particularly for high-risk populations such as the elderly. Remazolam’s ability to maintain stable hemodynamic parameters, such as blood pressure and heart rate, is critical in preventing cerebral perfusion impairment during surgery, a known risk factor for POCD.^[16–23]^ This stability positions remazolam as a safer option for elderly patients, who are more vulnerable to hemodynamic fluctuations.^[16,17,19,22]^ Additionally, remazolam’s rapid onset and shorter recovery time enhance patient comfort and healthcare efficiency, reducing the burden of prolonged sedation and allowing for faster turnover in both surgical and outpatient settings. The drug’s lower incidence of adverse reactions further underscores its favorable safety profile.^[19,20]^ This can be attributed to its targeted modulation of GABA receptors, which provides effective sedation while minimizing excessive neuronal suppression, making it particularly beneficial for elderly patients prone to sedative-related complications.

Moreover, the study suggests that remazolam may have neuroprotective properties by mitigating neuroinflammation and oxidative stress, key contributors to POCD pathogenesis.^[18,20]^ The observed reductions in inflammatory markers, such as IL-6 and S100β, indicate its potential to protect against cognitive decline. These findings suggest that remazolam offers benefits beyond sedation, potentially addressing the underlying mechanisms of POCD.^[18,20]^ However, further research is required to confirm these effects and elucidate the pathways involved. The combination of hemodynamic stability, reduced inflammatory response, and rapid recovery underscores remazolam’s potential as a versatile anesthetic agent across various surgical contexts.^[16–19,21–23]^ Its application should be tailored to individual patient needs, taking into account factors such as baseline cognitive status, comorbidities, and specific procedural requirements.

Despite these promising results, the study is not without limitations. The relatively small sample size and the lack of long-term follow-up data may limit the generalizability of the findings. Larger, multicenter trials with extended follow-up periods are needed to validate these results and assess the sustained cognitive effects of remazolam. Future research should also explore its interactions with other anesthetic agents and its role in multimodal strategies for preventing POCD. Additionally, investigating optimal dosing regimens and expanding its use in diverse surgical populations could provide further insights into its clinical utility.

## 7. Summary

This study evaluates the impact of remazolam on POCD, a significant condition that impairs memory, attention, and executive function, particularly in elderly surgical patients. Compared to traditional sedatives like midazolam, remazolam demonstrates several key advantages, including a rapid onset of action, faster recovery times, and fewer adverse reactions, making it a promising option for perioperative sedation. One of the most notable benefits is its superior hemodynamic stability, which ensures consistent blood pressure and heart rate during surgery. This stability helps preserve cerebral perfusion, reducing the risk of intraoperative hypoperfusion, a critical factor in preventing POCD. Furthermore, remazolam exhibits potential neuroprotective effects by reducing inflammatory markers such as IL-6 and S100β, which are implicated in neuroinflammation and oxidative stress, key contributors to the pathogenesis of POCD. These findings suggest that remazolam offers both safety and efficacy, particularly for elderly and high-risk populations, where minimizing cognitive complications is a priority. However, the study is limited by a small sample size and the lack of long-term follow-up data, which may constrain the generalizability of the results. Future research should involve larger, multicenter trials with extended follow-up to validate these findings and explore remazolam’s efficacy in diverse patient populations.

## Author contributions

**Conceptualization:** Liang Zhang, Qing-Hua Wang, Yi Qiu, Yumei Ding, Liang-Liang He.

**Data curation:** Liang Zhang, Liang-Liang He.

**Funding acquisition:** Liang Zhang, Yi Qiu, Yumei Ding, Xiao-Dong Wang, Zhi-Feng Zhang.

**Investigation:** Liang-Liang He.

**Methodology:** Liang Zhang, Qing-Hua Wang.

**Project administration:** Liang-Liang He.

**Resources:** Liang Zhang, Qing-Hua Wang, Yi Qiu, Xiao-Dong Wang, Zhi-Feng Zhang, Yi-Fan Zhao.

**Supervision:** Liang-Liang He.

**Validation:** Liang Zhang, Qing-Hua Wang, Yi Qiu, Yumei Ding, Xiao-Dong Wang, Yi-Fan Zhao, Liang-Liang He.

**Visualization:** Liang Zhang, Qing-Hua Wang, Yi Qiu, Yumei Ding, Xiao-Dong Wang, Zhi-Feng Zhang, Yi-Fan Zhao, Liang-Liang He.

**Writing – original draft:** Liang Zhang, Qing-Hua Wang, Yi Qiu, Yumei Ding, Xiao-Dong Wang, Zhi-Feng Zhang, Yi-Fan Zhao, Liang-Liang He.

**Writing – review & editing:** Liang Zhang, Qing-Hua Wang, Yi Qiu, Yumei Ding, Xiao-Dong Wang, Zhi-Feng Zhang, Yi-Fan Zhao, Liang-Liang He.
